# Exercise and physiotherapy for enhancing muscle strength, managing lower-limb lymphedema, and improving sexual function in the treatment and survivorship of gynecological cancers

**DOI:** 10.3389/fonc.2026.1806464

**Published:** 2026-05-20

**Authors:** Yun Huang, YaoChuan Zhang

**Affiliations:** 1 School of Physical Education, Zhengzhou Normal University, Zhengzhou, Henan, China; 2 College of Physical Education and Health, Zhaoqing University, Zhaoqing, Guangdong, China

**Keywords:** exercise, gynecological cancers, lower-limb lymphedema, muscle strength, physiotherapy, sexual function

## Abstract

Gynecological neoplasms, encompassing ovarian, cervical, and endometrial cancers, frequently lead to considerable physical and functional disabilities as a result of surgical intervention, radiotherapy, and pharmacological treatments such as chemotherapy. Prominent among the most prevalent sequelae are muscular weakness, lymphedema of the lower extremities, and sexual dysfunction, which together undermine quality of life and long-term survivorship. Recent scholarly evidence underscores the significance of exercise and physiotherapeutic interventions in alleviating these detrimental consequences. Resistance training and multimodal exercise regimens have demonstrated efficacy in augmenting muscular strength, thereby enhancing functional autonomy and overall physical capability in survivors of gynecological malignancies. Interventions grounded in physiotherapy, which encompass lymphatic drainage techniques and specialized rehabilitation programs, are effective in the management of lower-limb lymphedema, leading to a reduction in swelling, discomfort, and the potential for secondary complications. Moreover, rehabilitation of the pelvic floor and regimented physical activity can exert a beneficial effect on sexual function, particularly in relation to arousal, orgasmic dysfunctions, and concomitant discomfort. Preclinical investigations further substantiate the mechanistic framework underlying these interventions, revealing advancements in muscle morphology, lymphatic flow, and neuromuscular capabilities. Notwithstanding these encouraging results, the variability in intervention methodologies and outcome assessment presents a significant constraint. This review amalgamates the existing clinical and preclinical literature regarding exercise and physiotherapeutic approaches, underscoring their combined efficacy in augmenting muscular strength, managing lymphedema, and enhancing sexual health within the context of gynecologic oncology. Such revelations may pave the way for future research endeavors and facilitate the formulation of individualized, multimodal rehabilitation strategies aimed at optimizing survivorship outcomes.

## 1 Introduction

Gynecological malignancies, encompassing ovarian, cervical, and endometrial cancers, represent a significant fraction of cancer diagnoses among women globally and present considerable challenges to health and overall quality of life (QoL) throughout the continuum of survivorship. These malignancies often necessitate comprehensive treatment modalities including surgical intervention, chemotherapy, and radiotherapy, while enhancing survival rates, frequently lead to enduring physical disabilities and functional constraints. Treatment-related sequelae, such as Sarcopenia and generalized deconditioning, contribute to a gradual decline in muscle strength, whereas surgical interference with pelvic lymphatic networks is a critical risk factor for the development of lower-limb lymphedema (LLL), distinguished by symptoms such as swelling, a sense of heaviness, and compromised mobility that adversely affect daily functioning and psychological health. Moreover, sexual dysfunction manifested through symptoms including dyspareunia, diminished arousal, difficulties with lubrication, and disorders of orgasm, is frequently reported by survivors of gynecological cancers, particularly following pelvic radiotherapy and extensive surgical procedures, yet it remains insufficiently addressed within clinical practice (1–3).

These physiological impairments collectively hinder autonomy and overall QoL, often interacting in multifaceted manners such that reduced muscle strength and mobility intensify the effects of lymphedema and sexual dysfunction on daily activities (4, 5). Despite the growing acknowledgment of the therapeutic efficacy of exercise and physiotherapy within the field of oncology, the extant literature pertaining to gynecologic cancer remains disjointed. For instance, the systematic scoping review conducted by Rose et al. (4) highlighted enhancements in muscular strength and physical functioning subsequent to exercise interventions; however, it did not prioritize LLL or sexual function as primary outcomes, nor did it incorporate mechanistic insights or rehabilitation principles across various domains. In a similar vein, a comprehensive review conducted by Hsu et al. (5) determined that physical exercise is both feasible and safe for the management of LLL; however, it acknowledged a limited clinical impact on volumetric changes or symptomatic relief and failed to address specific rehabilitation strategies aimed at enhancing strength or sexual health. Furthermore, narrative syntheses such as that by Wang et al. (6) investigated extensive mechanistic associations between physical activity and outcomes related to gynecologic cancer, yet did not systematically assess rehabilitation outcomes specific to particular domains. Concerning sexual health, systematic reviews authored by Brennen et al. (1) and Barcellini et al. (2) suggest that pelvic floor muscle training (PFMT) and conservative pelvic interventions may have beneficial effects on sexual function among survivors; however, these reviews are constrained by the variability in interventions and outcome measurements and do not consolidate effects across the domains of muscle strength and lymphedema. Existing meta-analyzes within the field of gynecologic oncology have predominantly elucidated the effects of rehabilitation interventions on overarching outcomes such as overall physical activity levels and QoL; however, they fail to delineate the specific impacts on muscle strength, LLL, and sexual function within a comprehensive framework (7). As a result, significant knowledge gaps remain concerning the manner in which exercise and physiotherapy interventions ought to be customized to effectively address these particular functional domains — and the rationale behind why such targeted methodologies may yield distinct advantages in comparison to generalized exercise recommendations.

Therefore, this review intends to meticulously assess and integrate the body of evidence pertaining to exercise and physiotherapy interventions, with a concentrated emphasis on the enhancement of muscle strength, the management of LLL, and the amelioration of sexual function in women diagnosed with gynecologic malignancies. By amalgamating both clinical findings and underlying mechanistic insights, this endeavor aspires to reconcile disparate bodies of literature and to develop a rehabilitation framework that is specific to this domain. This methodology transcends existing literature reviews by investigating the interconnected pathways that link muscle, lymphatic, and pelvic floor functionalities, scrutinizing the characteristics of interventions (including type, dosage, and oversight), and pinpointing customized strategies for effective clinical application. Functional deficits in muscular strength, lymphatic functionality, and sexual well-being are common yet interconnected consequences of gynecologic cancer therapies; however, these aspects remain inadequately addressed within conventional rehabilitation paradigms. There exists a critical necessity to translate mechanistic understandings into practical, individualized interventions that can be operationalized within survivorship care to enhance functional capacity, overall QoL, and long-term health outcomes.

## 2 Methods

A thorough examination of existing literature was performed utilizing various electronic databases, including but not limited to PubMed, Scopus, Web of Science, and Embase, covering the period from their inception until January 2026. The search methodology integrated relevant keywords and Medical Subject Headings (MeSH) pertinent to gynecologic malignancies (for instance, “ovarian cancer,” “cervical cancer,” “endometrial cancer”) with terminologies associated with exercise and physiotherapy interventions (such as “resistance training,” “physical therapy,” “rehabilitation,” “manual lymphatic drainage”) alongside outcome measures of significance, specifically muscle strength, LLL, and sexual function. Boolean operators (“AND,” “OR”) were employed to enhance the specificity of searches, and the reference lists of pertinent articles were meticulously reviewed to unearth additional qualifying studies. The following search string was used in PubMed: (“exercise” OR “physical activity” OR “physiotherapy” OR “rehabilitation”) AND (“gynecological cancers” OR “cervical cancer” OR “ovarian cancer” OR “endometrial cancer”) AND (“muscle strength” OR “lower-limb lymphedema” OR “lymphedema management” OR “sexual function” OR “sexual health”) AND (“survivorship” OR “cancer treatment” OR “rehabilitation outcomes”).

Research studies were eligible for inclusion if they involved adult females diagnosed with gynecologic malignancies at any stage of progression, assessed exercise or physiotherapy interventions aimed at addressing at least one of the three specified outcomes, and provided clinical or preclinical metrics, encompassing functional, physiological, or mechanistic outcomes. The criteria for exclusion encompassed non-English language publications, abstracts lacking full text, case reports, systematic reviews, and investigations that concentrated exclusively on pharmacological treatments devoid of an exercise or physiotherapy element. Identified studies underwent an independent screening process conducted by two reviewers, utilizing titles, abstracts, and full texts as criteria, with any discrepancies resolved through a consensus approach. Studies deemed eligible were categorized as clinical, encompassing randomized controlled trials, pilot studies, feasibility studies, and observational studies, or as preclinical, which included *in vitro* and animal studies exploring the mechanistic effects of exercise or physiotherapy. Essential data were extracted employing a standardized form, which included parameters such as study design, sample size, cancer type, intervention specifics, duration, outcome measures, and principal findings. The data were synthesized in a narrative format and organized according to the three principal outcome domains, while mechanistic insights derived from preclinical studies were incorporated to yield a holistic understanding of how interventions affect functional outcomes. Quantitative findings, including effect sizes and percentage improvements, were summarized whenever feasible to facilitate comparisons across the various studies.

## 3 Muscle strength impairment, lower-limb lymphedema, and sexual dysfunction in gynecological cancers: pathophysiology and clinical burden

**Figure 1:**
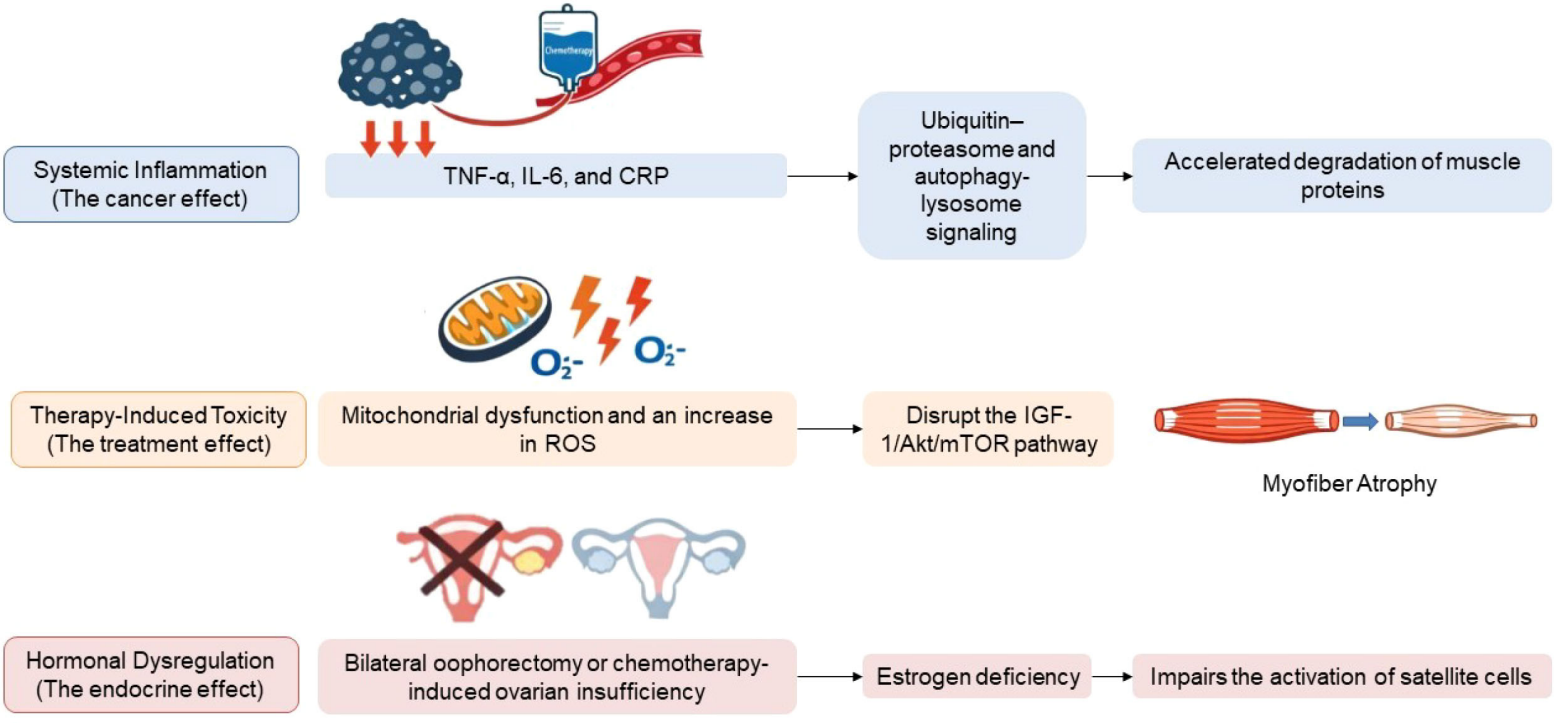
Etiological and molecular mechanisms underlying muscle strength impairment in gynecological cancer patients. This schematic illustrates how cancer-related systemic inflammation, treatment-induced toxicity, and hormonal dysregulation converge at the skeletal muscle level to promote muscle wasting. Tumor-driven inflammatory mediators and therapy-related oxidative stress activate proteolytic pathways while suppressing anabolic signaling, whereas estrogen deficiency impairs satellite cell–mediated regeneration. The integration of increased protein degradation, reduced protein synthesis, and defective repair culminates in myofiber atrophy, clinically manifesting as lower-limb weakness, impaired postural stability, reduced mobility, and early fatigue.

Gynecological neoplasms including cervical, ovarian, endometrial, vulvar, and vaginal cancers, are linked to significant and enduring functional disabilities that extend well beyond the conclusion of oncological intervention. Progress in early detection and integrated therapeutic approaches has markedly enhanced survival outcomes; nonetheless, survivorship is often complicated by persistent musculoskeletal, lymphatic, and sexual health issues. Among these complications, impairments in muscular strength, LLL, and sexual dysfunction are particularly prevalent, interrelated, and frequently inadequately addressed sequelae that considerably diminish physical functionality, psychological health, and overall QoL (**Figure 1**).

### 3.1 Muscle strength impairment in gynecological cancer patients

#### 3.1.1 Etiological and molecular mechanisms

The impairment of muscle strength observed in patients with gynecological malignancies is a consequence of a multifaceted interaction between systemic effects related to cancer, toxicity induced by therapeutic interventions, and behavioral determinants such as extended periods of physical inactivity. Systemic inflammation, characterized by elevated levels of circulating cytokines including tumor necrosis factor-α, interleukin-6, and C-reactive protein, triggers proteolytic mechanisms such as the ubiquitin–proteasome system and autophagy, lysosome signaling, culminating in the accelerated degradation of muscle proteins (8–10). The administration of chemotherapy further intensifies muscular wasting through the induction of mitochondrial dysfunction, augmented production of reactive oxygen species, and the disruption of insulin-like growth factor-1 (IGF-1)/Akt/mTOR signaling pathways, which ultimately leads to diminished muscle protein synthesis and atrophy of myofibers (11–13). Furthermore, radiotherapy targeting the pelvic region results in localized fibrosis, damage to microvasculature, and disruption of neuromuscular junctions, with a pronounced impact on the hip flexors, gluteal muscles, and musculature of the pelvic floor (14). Hormonal dysregulation subsequent to bilateral oophorectomy or chemotherapy-induced ovarian insufficiency is pivotal in the deterioration of muscle tissue. The deficiency of estrogen adversely influences the activation of satellite cells, the processes of muscle regeneration, and neuromuscular coordination (15, 16), thus expediting sarcopenic developments in both pre- and postmenopausal women.

#### 3.1.2 Clinical manifestations and functional consequences

In clinical contexts, muscle weakness manifests as diminished strength in the lower limbs, compromised postural stability, slowed ambulation, and an accelerated onset of fatigue (17). These deficits hinder the performance of activities of daily living, elevate the risk of falls, and restrict engagement in physical exercise and rehabilitation interventions. Significantly, the weakening of lower-limb and pelvic floor musculature disrupts the muscle pump mechanism, which is crucial for both venous and lymphatic return, thereby facilitating the onset and exacerbation of LLL (18, 19).

### 3.2 LLL in gynecological cancers

#### 3.2.1 Pathophysiology and risk factors

LLL constitutes a chronic and progressive pathology that arises from the disruption of the lymphatic system subsequent to pelvic lymph node dissection and/or radiotherapy. The excision or irradiation of lymph nodes diminishes the capacity for lymphatic transport, resulting in the accumulation of protein-rich interstitial fluid within subcutaneous tissues (20). Radiation-induced fibrosis exacerbates the impairment of lymphatic vessel contractility and lymphangiogenesis by modulating transforming growth factor-β signaling and the remodeling of the extracellular matrix. The sustained presence of lymph stasis incites chronic inflammation, macrophage infiltration, adipocyte hypertrophy, and advancing tissue fibrosis, which may culminate in the transformation of lymphedema into an irreversible state if left untreated (21, 22). The risk factors associated with LLL include the magnitude of lymphadenectomy, the application of combined chemoradiotherapy, obesity, postoperative infections, and diminished levels of physical activity. The incidence of LLL among gynecological cancer survivors varies between 20% and 47%, with elevated frequencies noted particularly in cases of cervical and vulvar cancers (23).

#### 3.2.2 Clinical and psychosocial impact

LLL presents as an accumulation of fluid in the extremities, accompanied by sensations of heaviness, discomfort, diminished joint flexibility, and recurrent episodes of cellulitis. These manifestations considerably disrupt gait mechanics and equilibrium, consequently restricting physical capabilities and intensifying muscular deconditioning. Beyond the realm of physical impairment, LLL is profoundly correlated with dissatisfaction regarding body image, as well as heightened levels of anxiety, depression, and social estrangement, particularly among younger individuals who have survived this condition (24).

### 3.3 Sexual dysfunction in gynecological cancer survivors

#### 3.3.1 Physiological mechanisms

Sexual dysfunction among patients with gynecological malignancies is a complex phenomenon characterized by a multitude of contributing factors, which include anatomical, neurovascular, hormonal, and musculoskeletal changes. Surgical procedures may compromise autonomic nerves that are integral to the physiological process of genital arousal, whereas pelvic radiotherapy can lead to the development of vaginal fibrosis, stenosis, diminished elasticity, and mucosal atrophy, thereby culminating in dyspareunia and a decline in sexual satisfaction (25, 26). The absence of estrogen plays a significant role in the reduction of vaginal lubrication, the atrophy of the epithelial layer, modifications in vaginal microbiota, and an increased vulnerability to inflammatory processes and pain (27). Furthermore, either weakness or hypertonicity of the pelvic floor muscles disrupts the typical sexual response by negatively affecting vaginal tone, perfusion, and the ability to achieve orgasm (28).

Brachytherapy, while essential for achieving optimal local control in gynecologic malignancies, is associated with a distinct profile of late toxicities, particularly affecting vaginal health and sexual function. Due to the high localized radiation doses delivered directly to the tumor and surrounding tissues, brachytherapy significantly contributes to structural and functional alterations of the vaginal canal. Among these, vaginal stenosis represents one of the most prevalent complications, characterized by fibrosis, reduced elasticity, and shortening of the vaginal length. These changes are strongly associated with dyspareunia, decreased lubrication, and difficulties with sexual intercourse, ultimately leading to reduced sexual activity and impaired quality of life (29, 30).

In addition to structural changes, brachytherapy may induce mucosal atrophy, decreased vascularization, and chronic inflammation, further exacerbating vaginal dryness and discomfort. Clinical studies have also reported alterations in sexual response, including decreased arousal and orgasmic function, which may be influenced by both local tissue damage and psychological factors related to cancer treatment. Importantly, the severity of these toxicities has been shown to correlate with radiation dose distribution and treatment parameters, underscoring the need for careful planning and dose optimization (31).

Preventive and therapeutic interventions are therefore critical. Vaginal dilator therapy (VDT) is widely recommended following brachytherapy to maintain vaginal patency and reduce the risk of adhesions, with evidence suggesting improved long-term sexual outcomes in adherent patients (32). Adjunctive strategies, including topical estrogen therapy (when appropriate) and sexual counseling, may further support recovery of sexual function. Early patient education and structured follow-up are essential to improve adherence to these interventions and to address the often-underreported impact of brachytherapy on sexual health.

Pelvic radiotherapy remains a cornerstone in the management of gynecologic malignancies and is typically delivered as a combination of external beam radiotherapy (EBRT) and brachytherapy, particularly in the curative treatment of cervical cancer and in adjuvant settings. The integration of brachytherapy allows the delivery of high, localized radiation doses to the tumor while sparing surrounding tissues; however, it is also associated with significant dose-dependent toxicity affecting adjacent normal structures (33). Among the most clinically relevant late adverse effects are vaginal, pelvic floor, and lymphatic complications, which substantially impact quality of life.

Radiation-induced vaginal stenosis is one of the most common sequelae of pelvic radiotherapy and brachytherapy, resulting from progressive fibrosis, reduced vascularization, and tissue hypoxia. These pathophysiological changes lead to narrowing, shortening, and decreased elasticity of the vaginal canal, often associated with dyspareunia, reduced lubrication, and impaired sexual function (29, 30). The risk and severity of vaginal stenosis are strongly correlated with total radiation dose and the volume of irradiated vaginal tissue, factors particularly relevant in brachytherapy due to its high localized dose distribution (31). In addition to vaginal toxicity, radiation-induced damage to pelvic floor musculature and neural structures may result in pelvic floor dysfunction, including urinary and fecal incontinence, while lymphatic injury contributes to the development of chronic lymphedema and associated functional limitations.

Given these multifactorial impairments, early and targeted rehabilitation strategies are essential. VDT is widely recommended as a first-line preventive and therapeutic intervention for vaginal stenosis, with consensus guidelines supporting its use following pelvic radiotherapy and brachytherapy (32). Regular dilator use has been associated with improved vaginal patency and sexual function, although adherence remains a significant clinical challenge (31). Additional supportive measures include topical therapies, such as vaginal estrogen (when not contraindicated), to improve mucosal integrity and reduce atrophy. Pelvic floor muscle training, often combined with biofeedback, has demonstrated benefits in improving muscle strength, coordination, and continence outcomes, addressing both functional and sexual sequelae of treatment.

Furthermore, the management of radiation-induced lymphedema requires specialized interventions, including manual lymphatic drainage, compression therapy, and exercise-based rehabilitation. Importantly, these interventions should be delivered within a multidisciplinary framework involving gynecologic oncologists, radiation oncologists, physiotherapists, and sexual health specialists. Such an approach facilitates early identification of treatment-related toxicities and enables timely initiation of individualized rehabilitation programs.

Overall, the incorporation of structured rehabilitation strategies into standard oncologic care is crucial for mitigating the long-term morbidity associated with pelvic radiotherapy and brachytherapy. Greater emphasis on patient education, early intervention, and adherence to rehabilitation protocols may significantly improve functional outcomes and quality of life in this patient population.

Pelvic radiotherapy is a major contributor to long-term morbidity in gynecological cancer survivors, with well-documented effects on vaginal, pelvic floor, and lymphatic structures. Radiation-induced fibrosis and reduced tissue elasticity can lead to vaginal stenosis, decreased lubrication, and dyspareunia, significantly impairing sexual function. In addition, damage to pelvic floor musculature and neural structures may result in pelvic floor dysfunction, including urinary and fecal incontinence. Lymphatic injury following radiotherapy further contributes to chronic lymphedema and associated functional limitations (34).

Targeted rehabilitation strategies are essential to mitigate these complications. VDT and topical estrogen (when not contraindicated) are commonly recommended to prevent or manage vaginal stenosis and maintain tissue elasticity. Pelvic floor muscle training, often combined with biofeedback, can improve muscle strength, coordination, and continence outcomes. Furthermore, manual lymphatic drainage and compression-based therapies play a key role in the management of radiation-induced lymphedema (34).

A multidisciplinary rehabilitation approach, involving physiotherapists, gynecologists, and oncology specialists, is critical to address the complex and overlapping sequelae of radiotherapy. Early initiation of these interventions may improve long-term functional outcomes and quality of life in this patient population (34).

#### 3.3.2 Psychological and relational factors

Beyond physiological alterations, psychological distress constitutes a pivotal factor in the manifestation of sexual dysfunction. Modifications in body image, diminished fertility, apprehension regarding recurrence, and anxiety associated with cancer markedly compromise sexual desire and intimacy. Cultural stigmas, coupled with inadequate communication between clinicians and patients, exacerbate the underdiagnosis and insufficient treatment of sexual health issues within the realm of gynecological oncology (35, 36).

### 3.4 Interconnected pathophysiology of muscle weakness, lymphedema, and sexual dysfunction

The aforementioned triad of conditions signifies interrelated elements within a cohesive pathophysiological framework. Compromised muscle strength adversely affects lymphatic circulation and the integrity of the pelvic floor, thereby facilitating the onset of lymphedema and sexual dysfunction. Conversely, the pain and edema associated with lymphedema restrict mobility and diminish physical activity, which in turn exacerbates muscle atrophy. Concurrently, dysfunction of the pelvic floor contributes to a spectrum of urinary complications, sexual pain syndromes, and diminished core stability (37, 38). This intricate burden highlights the imperative for multimodal exercise and physiotherapeutic strategies aimed at enhancing skeletal muscle strength, promoting lymphatic drainage, and optimizing pelvic floor function as essential components of gynecological cancer prevention, therapeutic intervention, and survivorship care.

## 4 Exercise and physiotherapy for LLL

**Figure 2:**
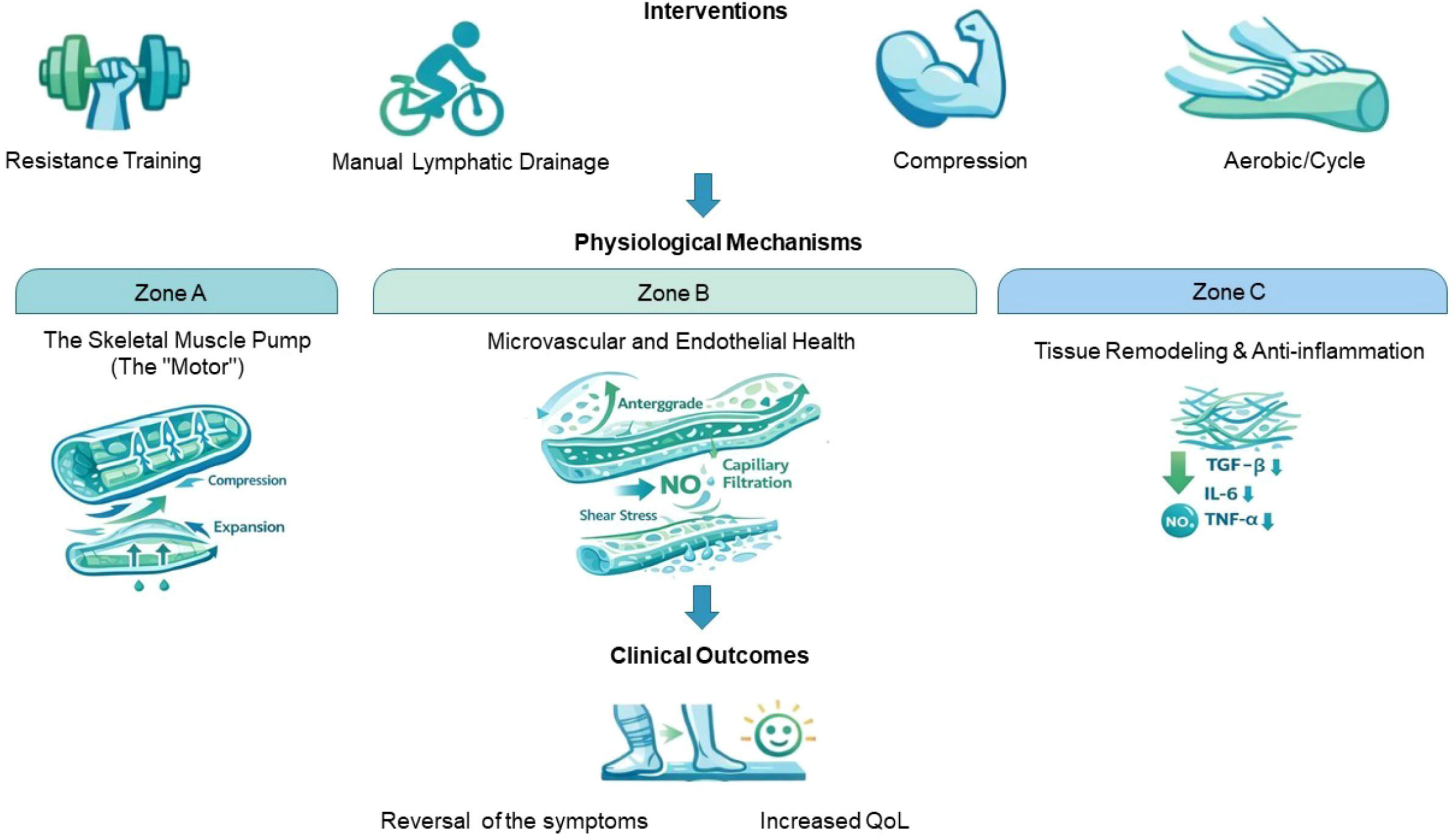
Biological mechanisms of exercise-induced rehabilitation targeting lower-limb lymphedema and muscle atrophy. This figure depicts the mechanical-to-biological translation of exercise-based interventions, including resistance training, aerobic activity, manual lymphatic drainage, and compression therapy. Muscle contractions activate the skeletal muscle pump to enhance anterograde lymphatic flow, while shear stress improves endothelial function via nitric oxide–mediated vasodilation. Concurrently, exercise-driven tissue remodeling reduces fibrosis and inflammatory signaling. These coordinated mechanisms lead to reduced limb volume, improved neuromuscular control, and enhanced quality of life.

LLL constitutes a debilitating and frequently chronic complication arising from the treatment of gynecologic malignancies (39, 40), particularly following extensive pelvic lymphadenectomy, with a cumulative incidence that may approach fifty percent of survivors within a decade post-surgery. This condition is defined by enduring edema, discomfort, a sensation of heaviness, and alterations in tissue integrity that collectively result in compromised mobility, decreased engagement in quotidian activities, as well as psychosocial disturbances such as anxiety, depression, and a diminished QoL. While the management of lymphedema has conventionally focused on complex decongestive therapy (CDT) (41, 42), the incorporation of exercise in conjunction with compression, educational initiatives, and mobility-enhancing strategies shows potential; nevertheless, rigorous and adequately powered research remains imperative to elucidate the most effective exercise modalities, optimal timing, and appropriate dosing for LLL within the context of gynecologic oncology (**Figure 2**).

A randomized pilot investigation examined the impact of a complex rehabilitation (CR) regimen in conjunction with CDT on edema, physical functionality, and QoL in a cohort of 40 patients exhibiting unilateral LLL subsequent to gynecologic oncology surgery. Subjects were allocated randomly to receive either CDT exclusively (n=20) or a combination of CR and CDT (CRCDT; n=20). The CR regimen entailed stretching, strengthening, and aerobic exercises conducted for 40 minutes, five times per week over a four-week duration, whereas CDT was initially administered intensively by a licensed physical therapist and subsequently by the patients themselves. Both groups evidenced statistically significant enhancements in edema, fatigue, pain, and lymphedema-specific symptoms. Nevertheless, the CRCDT cohort exhibited more pronounced improvements in physical functionality, quadriceps strength, fatigue alleviation, and chair-stand performance when compared to those receiving CDT alone. The findings of the study suggested that the integration of CR with CDT markedly augments muscular strength and functional outcomes without exacerbating edema in survivors of gynecologic cancer (43) (**Table 1**). Another study assessed the effectiveness of early CDT in conjunction with rehabilitative exercises for mitigating the onset of postoperative LLL in a cohort of 109 women who underwent surgical intervention for gynecologic malignancies. Participants were systematically allocated to either standard care (control) or the CDT regimen complemented by rehabilitative exercises, which encompassed professional education and comprehensive hip joint mobility exercises. Evaluative measures included the incidence of lymphedema, limb circumference, patient-reported symptoms (such as heaviness, pain, numbness, and dysfunction), QoL metrics (as measured by the EORTC QLQ-C30), and cancer-associated fatigue (utilizing the Brief Fatigue Inventory). The occurrence of lymphedema was statistically significantly lower among the CDT cohort (15.09%) in comparison to the control group (32.14%), with an extended duration of lymphedema-free survival observed. Participants receiving CDT also indicated a decrease in limb heaviness and pain, a reduction in limb diameters, enhancements in both overall and functional QoL scores, as well as diminished fatigue levels. The results of this study indicated that the early application of CDT alongside rehabilitative exercises significantly mitigates the risk of lymphedema while concurrently improving physical function, QoL, and fatigue outcomes in patients diagnosed with gynecologic cancer (44).

**Table 1:** Effects of exercise or physiotherapy interventions on lower-limb lymphedema in gynecological cancers.

Population	Intervention	Duration	Main outcomes	Key findings	Study
40 patients with unilateral lower-limb lymphedema	CDT vs. CDT + exercise rehabilitation	4 weeks	Edema, physical function, QoL	Improved edema and symptoms; CDT+CR showed greater gains in strength, physical function, and fatigue	(43)
109 postoperative gynecologic cancer patients	Routine care vs. early CDT + exercise	–	Lymphedema incidence, limb size, QoL, fatigue	Lower lymphedema incidence, reduced limb symptoms, improved QoL and fatigue in CDT group	(44)
120 cervical cancer patients	Modified CDT vs. education only	–	Lymphedema incidence and volume	Significantly reduced incidence, lower excess volume, delayed onset	(45)
74 gynecological cancer survivors with lymphedema (≥5 years cancer-free)	Bandage therapy vs. FIR + bandage	–	Limb fluid, circumference, safety	FIR group showed greater reduction in tissue fluid and limb size; no increase in CA125, recurrence, or adverse effects	(46)
44 patients with LLL after pelvic cancer treatment	Intensive CDP	–	Excess limb volume	Significant reduction in excess volume (−55.1%)	(47)
213 uterine cancer survivors	Physical activity and walking	–	LLL prevalence	Higher physical activity and walking associated with lower odds of LLL	(48)
60 gynecologic cancer patients post-surgery	Elastic-band resistance vs non-resistance exercise	–	Limb circumference, QoL, self-management	Both improved physical and global health scores	(49)
63 patients with lower extremity lymphedema	CDT vs CDT + cycling	3 weeks	Limb volume, function, QoL	Cycling group showed greater volume reduction and functional and QoL gains	(50)
24 cervical cancer patients post-radical hysterectomy	Progressive resistance exercise	24 weeks	Feasibility, safety, adherence	High adherence, no adverse events, acceptable difficulty	(51)
21 patients with lower-limb lymphedema	HIIT cycling with vs without compression	–	Safety, symptoms, limb volume	no clinically relevant volume or symptom differences	(52)
72 patients with LLL after endometrial or cervical cancer	CDT vs no treatment	–	Proprioception, balance, sensation	Improved knee proprioception and 2-point discrimination	(53)
267 cervical cancer patients	Resistance exercise vs compression vs control	–	LLL incidence	Lowest LLL incidence in exercise group (9.0%)	(54)
66 patients with secondary lower-limb lymphedema	MLD vs MLD + isokinetic strength training	4 weeks	Limb volume, gait, muscle strength	Greater volume reduction, improved walking ability, and increased muscle strength in combined group	(55)
100 gynecologic cancer patients after lymphadenectomy	Prophylactic physiotherapy vs education only	–	Lymphedema incidence, symptom scores	Lower lymphedema rate (5% vs 60%)	(56)

LLL, lower-limb lymphedema; CDT, complex decongestive therapy; CDP, complex decongestive physiotherapy; CR, complex rehabilitation; MLD, manual lymphatic drainage; FIR, far infrared radiation; HIIT, high-intensity interval training; QoL, quality of life; PA, physical activity; PRET, progressive resistance exercise training; PEV, percentage of excess volume; PREV, percentage reduction of excess volume; BIS, bioimpedance spectroscopy; DXA, dual-energy X-ray absorptiometry; LEFS, Lower Extremity Functional Scale; LYMQOL, Lymphedema Quality of Life Questionnaire; EORTC QLQ-C30, European Organization for Research and Treatment of Cancer Quality of Life Questionnaire C30; GCLQ, Gynecologic Cancer Lymphedema Questionnaire.

The aim of study was to determine the efficacy of modified complex decongestive physiotherapy (CDP) in the prevention of secondary LLL among a cohort of 120 cervical cancer patients undergoing laparoscopic radical hysterectomy accompanied by pelvic lymphadenectomy. Participants were systematically allocated to either the modified CDP intervention cohort (which included manual lymph drainage, compression hosiery, structured exercise regimens, and health education) or a control cohort receiving solely health education. A total of 117 patients successfully completed the one-year follow-up assessment. The prevalence of secondary LLL was markedly lower in the intervention cohort (13.6%) in comparison to the control cohort (34.5%), which corresponded with a reduced median percentage of excess limb volume (2.1% versus 2.96%) and a delayed onset of lymphedema (8.0 versus 4.6 months). These observations demonstrated that modified CDP constitutes an efficacious prophylactic intervention for averting postoperative LLL in cervical cancer patients, thereby advocating for the early incorporation of physiotherapy and structured exercise within the postoperative care framework (45). Another work evaluated the efficacy and safety of far infrared radiation (FIR) therapy in addressing gynecological cancer-related lymphedema (GCRL) among 74 women who had been cancer-free for a minimum duration of five years. Participants were randomly allocated to receive either conventional care involving bandaging or FIR in conjunction with bandaging. The clinical outcomes evaluated included limb circumference, tissue fluid accumulation, serum CA125 levels, and lymph node assessment across a one-year period. The FIR cohort exhibited statistically significant reductions in both limb fluid and circumference relative to the control group, with no observed increase in CA125 levels, recurrence of cancer, lymphadenopathy, or adverse effects. Complementary *in vitro* investigations revealed that FIR did not influence cell viability, proliferation, apoptosis, or cell cycle dynamics in fibroblasts and gynecologic cancer cell lines (A2780, SKOV-3, HeLa, Ishikawa). These data demonstrated that FIR represents a safe and effective adjunctive therapy for the management of lymphedema in survivors of gynecological cancer (46).

A retrospective investigation assessed the therapeutic efficacy of an intensive CDP program and identified predictors of treatment response in a cohort of 44 patients exhibiting LLL subsequent to pelvic cancer treatment. The majority of patients were diagnosed with cervical (61.4%), endometrial (20.5%), or ovarian cancer (18.2%), with an average age of 62.2 years. Participants engaged in an average of 12.6 daily CDP sessions, with an average duration of lymphedema lasting 34.8 months. The severity of lymphedema, quantified as the percentage of excess volume (PEV), demonstrated a statistically significant reduction from 32.9% ± 18.4% at baseline to 18.8% ± 16.7% post-treatment (p<0.001), which corresponds to a mean percentage reduction of excess volume (PREV) of 55.1%. Baseline PEV emerged as the sole predictor of CDP efficacy, whereas variables such as age, radiotherapy, lymphedema duration, and the number of sessions did not show predictive value. The study concluded that intensive CDP is efficacious for the management of LLL, and it is recommended that even patients presenting with mild lymphedema be referred for appropriate therapeutic intervention (47). A cross-sectional investigation evaluated the correlation between physical activity (PA), walking, and LLL in a cohort of 213 survivors of uterine cancer. Participants provided self-reported data regarding their PA quantified in MET-hours per week and their walking activity measured in blocks per day, in addition to reporting symptoms associated with LLL. Notably, 36% of the participants were categorized as exhibiting LLL. Elevated levels of PA were correlated with decreased odds of LLL in a dose-dependent fashion: individuals reporting ≥18 MET·h·wk exhibited an odds ratio of 0.32 in comparison to those reporting <3 MET·h·wk (P trend=0.003). Moreover, increased walking demonstrated a similarly protective effect: participants who walked ≥12 blocks per day presented an odds ratio of 0.19 for LLL relative to those walking <4 blocks per day (P trend <0.0001). The observed associations were moderated by body mass index (BMI) in relation to PA, but not in the context of walking. The findings of this study exhibited that higher levels of PA and walking may mitigate the risk of LLL in survivors of uterine cancer, thereby necessitating further prospective investigations to validate these results (48).

A randomized controlled trial examined the comparative effects of resistance exercises versus non-resistance exercises in a cohort of 60 patients who had undergone gynecological cancer surgery, with a focus on limb circumference, self-management of lymphedema, and overall QoL (QoL). Participants underwent assessments at baseline, one week following the intervention, and again three months’ post-intervention. The analysis revealed no statistically significant differences between the two groups regarding the incidence of lymphedema, limb circumference measurements, or lymphedema-specific QoL outcomes. Nevertheless, both exercise groups exhibited notable enhancements in general physical function, role function, and overall health status (β = 0.69–1.43) over the duration of the study. Additionally, there was a significant improvement in lymphedema self-management from baseline to the follow-up assessments. The findings of the study suggest that resistance training is as safe and practicable as non-resistance training for survivors of gynecologic cancer, does not aggravate lymphedema, and can be seamlessly incorporated into daily routines to facilitate physical function and preventive health care. Both modalities of exercise may play a role in mitigating the risk of developing LLL following surgical intervention (49). Another study assessed the efficacy of integrating aerobic exercise with CDT in a cohort of 63 patients suffering from lower extremity lymphedema (LEL) associated with gynecologic malignancies. Subjects were allocated randomly to either a CDT-only cohort (n=31) or a CDT in conjunction with cycle ergometry cohort (n=32). The cycling component entailed 20 minutes of moderate-intensity aerobic exercise performed on a stationary bike over the course of three weeks. Evaluated outcomes encompassed limb volume, lower extremity functionality (LEFS), and quality of life metrics (LYMQOL). Both cohorts exhibited statistically significant enhancements across all measured outcomes (P<0.001). Nevertheless, the CDT combined with cycling cohort manifested more pronounced decreases in limb volume and more substantial enhancements in functionality and various quality-of-life dimensions, such as function, appearance, symptoms, mood, and overall QoL (P = 0.002–0.04). The study indicated that the incorporation of aerobic exercise within the framework of CDT amplifies the therapeutic advantages for LEL, thereby fostering improvements in physical functionality and overall QoL among survivors of gynecologic cancers (50).

A preliminary investigation evaluated the practicality of progressive resistance exercise training (PRET) in mitigating the incidence of LLL among 24 patients diagnosed with cervical cancer who underwent radical hysterectomy accompanied by pelvic lymphadenectomy. Participants engaged in PRET bi-daily for a duration of 24 weeks, commencing with two weeks of supervised training within a hospital setting, followed by 22 weeks of exercise conducted in a home environment, with a follow-up period extending to 12 months. Measured outcomes encompassed limb volume, BMI, subjective exercise difficulty, adherence rates, and the occurrence of adverse events. None of the participants withdrew from the study, and adherence rates surpassed 75% for the majority of individuals. The perceived challenge associated with the exercises was generally deemed suitable, and no significant adverse events were reported. The investigation established that PRET is both safe and feasible, as well as well-accepted within the postoperative context. These results lay the groundwork for a more extensive randomized controlled trial aimed at assessing the preventative impacts of PRET on LLL in survivors of cervical cancer (51). A crossover pilot investigation assessed the safety and feasibility of high-intensity interval training (HIIT) conducted on a stationary bicycle among 21 individuals diagnosed with cancer-related LLL, both with and without the use of compression garments. Participants engaged in two training sessions that were separated by a seven-day washout period. Safety metrics encompassed adverse events and self-reported symptoms (such as pain, heaviness, and tension), while limb volume, soft tissue mass, and extracellular fluid were quantified through circumferential assessment, dual-energy X-ray absorptiometry (DXA), and bioimpedance spectroscopy. A total of nineteen participants (90%) successfully completed both training sessions, indicating a high level of acceptability. No adverse events were reported, and the exercise regimen did not exacerbate the symptoms or volume associated with LLL. Modest yet statistically significant enhancements in soft tissue mass (164.2 g, 1.4%) and extracellular fluid were noted when compression garments were utilized. The observations of this study shown that HIIT on a stationary bicycle is a safe, feasible, and well-accepted intervention for patients suffering from LLL, with compression garments potentially offering slight additional advantages (52).

The purpose of study was to evaluate the impact of CDT on proprioceptive acuity, balance capabilities, and sensory functionality in a cohort of 72 patients diagnosed with LLL subsequent to endometrial or cervical malignancies. Participants were allocated randomly to either a CDT intervention group (n=36) or a control cohort (n=36). Evaluative outcomes encompassed knee joint proprioception assessed at flexion angles of 15°, 45°, and 60°, single-leg balance performance with both eyes open and closed, light touch sensation, and two-point discrimination (2PD). The CDT intervention group exhibited statistically significant enhancements in knee proprioception across all measured angles (P<0.001) and in two-point discrimination (P = 0.012). Improvements in balance were noted within the CDT cohort; however, intergroup differences did not reach statistical significance. No notable alterations were detected in light touch sensation. The findings indicated that while CDT may not entirely rectify sensory deficits or balance impairments in patients with LLL, it does demonstrate efficacy in enhancing kinesthetic awareness and joint proprioception, thereby underscoring its importance in functional rehabilitation (53). An open-label randomized controlled trial systematically assessed the efficacy of a PRET protocol in mitigating the occurrence of LLL among 267 patients diagnosed with cervical cancer subsequent to radical surgical intervention. Participants were allocated randomly into three distinct groups: the PRET group (n=89), the graduated compression stockings group (n=89), and a control cohort (n=89), with a follow-up duration of two years. The prevalence of LLL was documented at 9.0% within the PRET cohort, 28.1% in the compression stockings cohort, and 42.7% in the control cohort. The likelihood of developing LLL was markedly diminished in the PRET group in comparison to the control group (HR 0.156, 95% CI 0.073–0.335). While the application of compression stockings suggested a potential benefit, the observed outcome did not achieve statistical significance. The study ultimately concluded that PRET constitutes a safe, convenient, cost-effective, and accessible intervention for the prevention of LLL following pelvic lymphadenectomy, thereby endorsing its implementation in postoperative rehabilitation, particularly in contexts characterized by limited resources (54). Another research investigated the impact of integrating isokinetic strength training with manual lymphatic drainage (MLD) in a cohort of 66 patients exhibiting secondary LLL subsequent to gynecologic cancer surgery. Participants were allocated randomly into a control group receiving MLD exclusively or an experimental group undergoing MLD in conjunction with 20 minutes of daily isokinetic strength training over a four-week period. The outcomes assessed encompassed limb volume (quantified via circumference), ambulation proficiency (evaluated using the Holden Gait Scale), and muscular strength (assessed through Lovett grading). Baseline measurements were comparable across the groups. Following the intervention, the experimental group exhibited statistically significant reductions in limb volume, enhancements in ambulation proficiency, and increases in muscular strength when contrasted with the control group (P<0.05). The investigation concluded that the incorporation of isokinetic strength training into standard MLD protocols significantly augments the management of secondary LLL, thereby improving both functional mobility and muscular performance among survivors of gynecologic cancer (55).

The aim of study was to examine the effectiveness of prophylactic complex physiotherapy in mitigating the occurrence of LLL among a cohort of 100 patients diagnosed with gynecological malignancies who underwent lymphadenectomy. Participants were provided with pre-adjuvant physiotherapeutic interventions, encompassing both massage techniques and exercise regimens, whereas those who opted out were merely furnished with informational materials. The incidence of lymphedema, along with patient-reported symptoms—as measured by the Gynecologic Cancer Lymphedema Questionnaire—was evaluated at 1, 3, 6, and 12 months following surgical intervention. Lymphedema manifested in a mere 5% of individuals within the physiotherapy cohort, contrasting sharply with a 60% incidence rate observed in the non-physiotherapy group. The median symptom scores were notably lower in the physiotherapy group (3 versus 16), indicative of alleviated heaviness, pain, and functional impairment. Elevated lymphedema rates within the non-physiotherapy cohort were correlated with systematic lymphadenectomy and an increased volume of lymph node extraction. The findings of this study indicated that prophylactic complex physiotherapy is both safe and efficacious, significantly diminishing the incidence of LLL and the symptomatic burden experienced by survivors of gynecologic cancer (56). Overall, physical exercise and physiotherapeutic interventions significantly ameliorate LLL in survivors of gynecologic malignancies through a multitude of interrelated biological mechanisms. Resistance training and aerobic exercises enhance the functionality of the skeletal muscle pump, thereby facilitating lymphatic and venous return while concurrently diminishing the accumulation of interstitial fluid. Techniques such as manual lymphatic drainage and complex decongestive physiotherapy promote lymphatic transport and decrease tissue fibrosis, thereby augmenting the benefits of active movement. Furthermore, exercise contributes to the enhancement of endothelial function and capillary filtration, effectively preventing fluid stasis, while also improving muscle strength and joint mobility, collectively supporting limb functionality and alleviating sensations of heaviness or discomfort. In addition, the combination of exercise and physiotherapy exerts a favorable influence on inflammatory mediators, thereby reducing chronic inflammation associated with edema. These physiological mechanisms culminate in a reduction of limb volume, an enhancement of functional mobility, improved proprioceptive abilities, and an overall elevated QoL, thereby endorsing the incorporation of individualized, multimodal rehabilitation programs within the framework of survivorship care.

## 5 Exercise and physiotherapy for muscle strength

An investigation assessed the viability and outcomes of an integrated supervised and home-based exercise regimen during chemotherapy among women diagnosed with recurrent ovarian cancer. A total of thirty participants were enlisted from oncology clinics across Australia and engaged in a 12-week tailored program that encompassed low to moderate aerobic, resistance, core stability, and balance exercises for a minimum of 90 minutes weekly. The feasibility metrics indicated a recruitment rate of 63%, a retention rate of 70%, and a high adherence rate of 81%, with no reported exercise-related adverse events. On average, participants engaged in 196 minutes of physical activity weekly. Those individuals who successfully completed the intervention exhibited substantial enhancements in QoL, fatigue levels, mental health, muscular strength, and balance capabilities. Furthermore, exercise completers attained a higher relative dose intensity of chemotherapy when compared to noncompleters. Collectively, the findings shown that engaging in low to moderate exercise during chemotherapy is both feasible and advantageous for this demographic, thus necessitating further validation through randomized controlled trials (57) (**Table 2**). The objective of study was to explore the impact of supervised resistance training on various parameters such as muscular health, strength, physical functionality, QoL, and pelvic-floor functionality in survivors of advanced-stage ovarian cancer following their initial treatment regimen. A cohort of fifteen participants undertook resistance training sessions bi-weekly over a span of 12 weeks, which were conducted either within a clinical setting or through telehealth modalities. Evaluations encompassed assessments of muscle mass and density, muscular strength, physical functionality, QoL, and self-reported pelvic-floor symptoms. All participants successfully completed the intervention with a notable adherence rate (median 92%). Following the intervention, significant enhancements were noted in both whole-body and appendicular lean mass, muscle density, strength in both upper and lower body, performance on a 400-meter walking task, timed up-and-go assessment, as well as in the social and cognitive domains of QoL, whereas pelvic-floor symptoms remained statistically unchanged (58).

**Table 2:** Effects of exercise intervention on muscle strength in gynecological cancers.

Population	Duration	Intervention	Main outcomes	Key findings	Study
Women with recurrent ovarian cancer on chemotherapy (n=30)	12-week	supervised + home exercise, ≥90 min/week, low–moderate intensity	Feasibility, strength, balance, fatigue, QoL, chemo completion	Recruitment 63%, retention 70%, adherence 81%. Improved QoL, fatigue, mental health, strength, balance.	(57)
Advanced ovarian cancer post first-line treatment (n=15)	12-week	supervised resistance exercise, 2x/week	Muscle mass/density, strength, physical function, QoL, pelvic-floor	92% attendance. Improved lean mass, muscle density, strength, 400-m walk, TUG, social/cognitive QoL.	(58)
Stage III/IV ovarian cancer survivors (n=12)	12-week	supervised resistance exercise; compliance tracked	Muscle mass, strength, function, QoL	Median compliance 63%. Higher compliance → greater lean mass (+1.3 kg) and lower body strength (+50 kg). Lower compliance → better 400-m walk.	(59)
Breast or ovarian cancer survivors (n=10)	8-week	upper extremity fitness boxing, 3x/week	6MWT, chair stand, balance, scapular/grip strength, fatigue, QoL	Most improved 6MWT, chair stand, grip and trapezius strength. FACIT-F showed decreased pain, fatigue, and improved sleep.	(60)
Gynecological cancer survivors with dyspareunia (n=31)	12-week	multimodal pelvic floor physiotherapy (education, exercises with biofeedback, manual therapy, home exercises)	Pelvic floor morphometry and muscle function	Significant improvements in levator hiatal narrowing, muscle tone, stiffness, flexibility, coordination, and endurance.	(61)
Post-treatment endometrial cancer survivors (n=40)	10-week	home-based strength training, 2x/week	Lean mass, functional fitness, blood biomarkers, QoL	Improvements in chair sit-to-stand, arm curl, and 8-ft up-and-go. No changes in flexibility, 6-min walk, handgrip, blood biomarkers, or patient-reported outcomes.	(62)
Gynecological cancer survivors (n=160)		Resistance exercise vs control, post-treatment	QoL, cancer-related fatigue, BMI, body fat, muscle strength	Exercise group improved QoL, fatigue, body composition, and strength significantly.	(63)

6MWT, 6-Minute Walk Test; TUG, Timed Up-and-Go test; QoL, Quality of Life; FACIT-F, Functional Assessment of Chronic Illness Therapy–Fatigue; BMI, Body Mass Index.

A research evaluated the exercise dosage administered throughout a 12-week, supervised resistance training regimen and its implications for muscle health, functional capabilities, and overall QoL among survivors of advanced-stage ovarian cancer. A cohort of twelve women diagnosed with stage III or IV ovarian cancer engaged in the study, with exercise metrics encompassing adherence levels, session adaptations, and subjective assessments of exertion. Participants were stratified into compliance categories of lower (<63%) or higher (>63%). The median adherence rate was recorded at 63%, accompanied by a perceived exertion level of 13 (“somewhat hard”), while dose modifications were necessitated in 92.7% of the sessions. Both compliance groups demonstrated enhancements in muscle morphology, strength, and QoL metrics. Higher adherence correlated with significantly greater increases in lean mass (+1.3 kg vs. +0.5 kg) and lower body strength (+50 kg vs. +13 kg), whereas clinically significant improvements in 400-meter walking time were observed solely within the lower compliance group. The findings exhibited that even a lower dosage of flexible resistance exercise can confer substantial benefits to survivors of advanced-stage ovarian cancer (59). A preliminary investigation assessed the impact of an 8-week upper extremity (UE) fitness boxing regimen on functional outcomes and QoL in female patients diagnosed with breast or ovarian cancer. A total of ten participants took part in instructor-led sessions conducted three times weekly, with assessments administered before and after the intervention, including the 6-Minute Walk Test (6MWT), chair stand, balance evaluations, scapular and grip strength assessments, as well as fatigue-related QoL (FACIT-F) metrics. Nine participants successfully completed the program. The majority demonstrated functional enhancements: 77% exhibited an increase in 6MWT distance, 55% showed improvements in chair stand performance, 66% enhanced trapezius strength, and 55–66% improved grip strength. The FACIT-F outcomes indicated a reduction in pain, fatigue, and side effects related to treatment, along with improved sleep quality in 22–44% of participants. Notwithstanding the modest sample size and the constraints on generalizability, UE fitness boxing seems to be a safe and potentially effective intervention for augmenting strength, endurance, and QoL in women affected by breast or ovarian cancer (60).

A prospective interventional study was conducted to examine the impacts of multimodal pelvic floor physiotherapy on the morphometric characteristics and muscular functionality of the pelvic floor in a cohort of 31 survivors of gynecological malignancies suffering from dyspareunia. The participants engaged in a regimen of 12 weekly sessions that integrated educational components, pelvic floor exercises supplemented with biofeedback mechanisms, manual therapeutic techniques, and prescribed home exercise routines. Evaluations conducted at baseline and following treatment encompassed 3D/4D ultrasound imaging to assess pelvic floor morphometry, as well as dynamometric assessments to evaluate muscle tone, flexibility, stiffness, strength, coordination, and endurance. Subsequent to the treatment, notable enhancements were identified in morphometric parameters, including a reduction in the levator hiatal area by 14%, and improvements in muscle functionality, characterized by a decrease in tone by -0.4 N, a reduction in stiffness by -0.1 N/mm, alongside an increase in flexibility by +9 mm, coordination by +3 rapid contractions, and endurance by +683%·s. These findings demonstrated that multimodal pelvic floor physiotherapy is efficacious in augmenting pelvic floor structural integrity and functional capacity, thereby providing a mechanistic framework for alleviating dyspareunia among survivors of gynecological cancer (61). A preliminary investigation assessed the impact of a 10-week home-based resistance training regimen on physiological and functional outcomes among 40 individuals who had completed treatment for endometrial cancer. The participants, with a mean age of 61 years and elevated BMI, engaged in supervised exercise sessions twice a week for a duration of 10 weeks, followed by a 5-week observational period. The outcomes measured included dual-energy X-ray absorptiometry (DXA)-assessed lean mass, functional fitness metrics, blood biomarkers, and QoL indicators. Notable enhancements were recorded in functional assessments, comprising the 30-second chair sit-to-stand test, the 30-second arm curl test, and the 8-foot up-and-go test; conversely, no significant alterations were detected in flexibility, 6-minute walking distance, handgrip strength, blood biomarkers (including HbA1c and C-reactive protein), or self-reported outcomes relating to anxiety, depression, fatigue, or exercise self-efficacy. These findings indicated the feasibility of home-based resistance exercise and its potential to yield clinically significant enhancements in physical function within a brief duration for survivors of endometrial cancer (62).

A randomized controlled trial aimed to investigate the impact of resistance training on the QoL and fatigue levels among a cohort of 160 survivors of gynecological malignancies. Subjects were systematically allocated to either an exercise intervention group (n=80) or a non-exercise control group (n=80). The primary endpoints comprised QoL and cancer-associated fatigue, which were evaluated utilizing validated assessment instruments, whereas secondary endpoints encompassed measurements of body composition and muscular strength. Following the completion of the intervention, the exercise group exhibited statistically significant enhancements in QoL, fatigue reduction, body composition, and muscular strength in comparison to the control group (P<0.001). Variations in physical metrics, such as BMI and body fat percentage, demonstrated a significant correlation with the observed improvements in QoL and fatigue levels (P<0.001). These results suggest that resistance training may serve as a potent modality for alleviating cancer-related fatigue and augmenting QoL, thereby advocating for its integration into the supportive care framework for individuals recovering from gynecological cancer (63). Across these investigations, exercise interventions including resistance training, upper-extremity fitness boxing, and multimodal physiotherapy, exhibited consistent advantages for women diagnosed with gynecological or breast malignancies. Mechanistically, exercise enhanced muscle morphology (lean mass, density), strength, and functional performance through the augmentation of muscle contractility, flexibility, coordination, and endurance. These physiological adaptations were accompanied by a decline in muscle tone, stiffness, fatigue, and dyspareunia, as well as enhancements in balance, aerobic capacity, and scapular/grip strength. Furthermore, exercise influenced systemic and psychosocial outcomes, thereby improving QoL, cognitive and social functioning, and sleep, while facilitating treatment adherence and tolerance. Collectively, these findings indicate that personalized, supervised, or home-based exercise fosters musculoskeletal, neuromuscular, and functional recovery via physiological remodeling and psychophysiological adaptations in cancer survivors.

## 6 Exercise and physiotherapy for sexual function

A multicenter prospective investigation assessed the practicality, acceptability, and outcomes of a 12-week multimodal pelvic floor physical therapy intervention in a cohort of 31 survivors of gynecological cancers experiencing dyspareunia. The therapeutic regimen incorporated educational components, manual therapy, pelvic floor exercises supplemented with biofeedback, and home-based exercises, which included the utilization of dilators. The outcomes pertaining to feasibility were notably high, with 88% adherence to prescribed home exercises, 94% attendance at a minimum of 10 sessions, and a dropout rate of merely 3%. Following the intervention, participants exhibited statistically significant enhancements in pain intensity and quality, sexual function, pelvic floor symptoms, and overall QoL (P ≤ 0.044). Satisfaction with the treatment was markedly high (9.3/10), with 90% of participants indicating considerable improvement. The findings of study indicated for the feasibility, acceptability, and efficacy of multimodal pelvic floor therapy, thereby necessitating further validation through randomized controlled trials (64) (**Table 3**). Another work assessed the efficacy of integrating vaginal dilator (VD) utilization with pelvic floor muscle exercises (PFME) in a cohort of 28 women receiving radiotherapy for cervical cancer, with PFME being administered prior to treatment and followed up after six months. At the four-month mark subsequent to the conclusion of radiotherapy, 91% of participants either preserved or augmented the size of the VD, while 82% reported being sexually active. Compliance with VD utilization was observed to be notably high. Although certain limitations in emotional functioning were identified, the intervention demonstrated effectiveness in mitigating the risk of vaginal stenosis, thereby reinforcing the combined application of VD and PFME to sustain vaginal health, enhance sexual function, and improve overall QoL during the course of radiotherapy (65).

**Table 3:** Effects of exercise or physiotherapy intervention on sexual function in gynecological cancers.

Population	Duration	Intervention	Main outcomes	Key findings	Study
Endometrial and cervical cancer survivors with dyspareunia (n=31)	12-week	multimodal pelvic floor physical therapy (education, manual therapy, biofeedback-guided PFMT, home exercises with dilator)	Feasibility, pain, sexual function, pelvic floor symptoms, QoL	High feasibility (88% adherence, 94% attendance, 3% dropout). Significant improvements in pain, sexual function, pelvic floor symptoms, and QoL. High satisfaction	(64)
Cervical cancer patients undergoing radiotherapy (n=28)	initiated pre-RT, 6-month follow-up	Vaginal dilator plus pelvic floor muscle exercises	Vaginal stenosis, sexual activity, QoL	91% maintained/increased dilator size; 82% sexually active post-RTable	(65)
Gynecological cancer survivors with pelvic floor dysfunction (n=34)	4-week	pelvic floor rehabilitation program vs usual care	Pelvic floor strength, sexual function, QoL	Improved pelvic floor strength and sexual function and physical and sexual QoL	(66)
Gynecological cancer survivors with dyspareunia (n=31)	12-week	multimodal pelvic floor physical therapy (education, manual therapy, PFMT) with 1-year follow-up	Pain, sexual function, sexual distress, psychosocial outcomes, pelvic floor symptoms	Significant improvements in pain, sexual functioning, psychological outcomes, and pelvic floor symptoms were sustained at 1-year follow-up. No decline from post-treatment levels.	(67)
Irradiated cervical cancer patients with sexual dysfunction (n=15; 13 completed)	4×/week for 3 months	Noninvasive clitoral therapy device (vacuum-induced engorgement),	Sexual desire, arousal, lubrication, orgasm, satisfaction, pain; genital tissue health	Significant improvements across all sexual function domains (FSFI and DISF scores). Patients improved from severe dysfunction to normal cutoff range.	(68)

PFPT, pelvic floor physical therapy, PFMT, pelvic floor muscle training, PFRP, pelvic floor rehabilitation program, VD, vaginal dilator, PFME, pelvic floor muscle exercises, QoL, quality of life, FSFI, Female Sexual Function Index, DISF, Derogatis Interview for Sexual Functioning.

A randomized controlled trial examined the impact of a four-week pelvic floor rehabilitation program (PFRP) on pelvic floor functionality and QoL among 34 survivors of gynecological cancer. A cohort of seventeen patients participated in PFRP exercises, whereas the remaining seventeen were subjected to standard care. Out of the initial participants, twenty-four successfully completed the study. The PFRP cohort exhibited statistically significant enhancements in pelvic floor strength (MD = 14.22, P = 0.036), sexual function, and overall physical and sexual QoL in comparison to the control group. These observations demonstrated that a short-term PFRP can substantially improve pelvic floor function and QoL for gynecological cancer survivors, warranting additional exploration in larger-scale trials (66). A mixed-method investigation assessed the longitudinal impacts of a 12-week multimodal pelvic floor physical therapy (PFPT) intervention on a cohort of 31 gynecological cancer survivors experiencing dyspareunia. The intervention comprised educational components, manual therapeutic techniques, and PFMT exercises. Quantitative evaluations revealed statistically significant and sustained enhancements in pain, sexual function, sexual distress, body image, pain-related anxiety, pain catastrophizing, self-efficacy regarding intercourse, depressive symptomatology, and pelvic floor disorder manifestations (P ≤ 0.028) when comparing baseline measurements to one-year follow-up assessments. Qualitative interviews elucidated that participants esteemed the alleviation of pain, enhancement of sexual function, and reduction of urinary symptoms, attributing these improvements to a confluence of biological, psychological, and social transformations. Participants perceived adherence to the therapy as a critical factor influencing the resultant outcomes. The data exhibited that PFPT yields enduring and significant benefits for dyspareunia among gynecological cancer survivors (67).

A preliminary investigation assessed the effectiveness of a clitoral therapy apparatus (Eros Therapy) in a cohort of 15 cervical cancer patients who had undergone radiation therapy and experienced sexual dysfunction. The subjects utilized the device four times weekly for a duration of three months, incorporating it into foreplay and self-stimulation activities. Thirteen participants successfully completed the entirety of the study. At the three-month mark, noteworthy enhancements were documented in sexual desire, arousal, lubrication, orgasm, satisfaction, and pain. The median score on the Female Sexual Function Index demonstrated an increase from 17 to 29.4 (P<0.001), while the score on the Derogatis Interview for Sexual Functioning escalated from 46 to 95 (P<0.001), transitioning from a subnormal to a nearly normal level of sexual functioning. Gynecological examinations additionally revealed improvements in mucosal health and vaginal elasticity (68). In the context of the aforementioned investigations, interventions specifically targeting the pelvic floor, which encompass multimodal physical therapy, pelvic floor muscle exercises (PFME), comprehensive rehabilitation programs, and clitoral therapy, have demonstrated significant enhancements in sexual function, alleviation of pain, and overall QoL for survivors of gynecological and cervical malignancies. From a mechanistic standpoint, these interventions have been shown to augment pelvic floor muscle strength, coordination, flexibility, and endurance, while concurrently diminishing muscle tone, stiffness, and sensitivity to pain, thereby fostering the health and elasticity of vaginal tissue. These improvements were further substantiated by favorable neuromuscular adaptations, increased localized blood circulation, and the normalization of sexual arousal responses. Additionally, psychological and social determinants, including diminished pain catastrophizing, bolstered self-efficacy, and perceived adherence to therapeutic protocols, played a contributory role, culminating in enduring enhancements in functional, psychosexual, and quality-of-life outcomes.

## 7 Integrative discussion

The reviewed literature consistently indicates that exercise and physiotherapeutic interventions yield substantial advantages across various functional domains for individuals who have survived gynecologic cancer. Specifically, resistance and multimodal exercise regimens significantly enhance muscular strength, thereby improving physical performance, facilitating functional autonomy, and elevating overall QoL. In a similar vein, physiotherapy-based modalities, such as manual lymphatic drainage integrated with targeted exercises, effectively diminish LLL, alleviating swelling, discomfort, and restrictions in mobility. Furthermore, emerging evidence suggests that pelvic floor rehabilitation and systematic exercise protocols can enhance sexual function, encompassing arousal, orgasmic response, and sexual satisfaction. Mechanistically, these interventions manifest synergistic effects: resistance training and functional exercises promote muscle hypertrophy, enhance neuromuscular activation, and stimulate myokine signaling, which may facilitate improvements in vascular function and lymphatic flow, thereby indirectly aiding in the management of lymphedema. Pelvic floor strengthening, alongside exercise-induced augmentations in localized blood flow, may bolster neurovascular function and tissue elasticity, thereby contributing positively to enhancements in sexual function. Collectively, these observations underscore the intricate interrelationship among musculoskeletal, lymphatic, and sexual health in survivors of gynecologic cancer. The prevailing empirical evidence emphasizes the considerable promise inherent in multimodal, individualized rehabilitation frameworks that amalgamate resistance training, physiotherapeutic interventions targeting lymphedema, and strategies aimed at enhancing pelvic floor and sexual function. Nevertheless, substantial deficiencies persist, notably the variability in intervention protocols, the constraints posed by small sample sizes, and the inadequacy of prolonged follow-up assessments. Subsequent investigations ought to prioritize extensive, randomized controlled trials employing standardized outcome metrics to refine rehabilitation methodologies and formulate evidence-based directives aimed at enhancing survivorship outcomes.

## 8 Conclusions

This review highlights the paramount importance of exercise and physiotherapeutic interventions in optimizing functional outcomes for survivors of gynecologic malignancies. Empirical evidence indicates that well-structured resistance training and multimodal exercise regimens significantly augment muscular strength, thereby facilitating enhancements in functional autonomy, mobility, and comprehensive physical performance. Physiotherapeutic interventions, which encompass manual lymphatic drainage, compression therapy, and specialized exercise, are efficacious in the management of LLL, leading to a reduction in swelling, discomfort, and related mobility constraints. Furthermore, interventions aimed at the pelvic floor and sexual functionality including exercise-oriented rehabilitation, exhibit promising advancements in sexual arousal, orgasmic response, satisfaction, and tissue elasticity, thereby addressing a frequently underappreciated dimension of survivorship. Mechanistically, these interventions yield synergistic advantages: resistance and functional exercises promote muscle hypertrophy, neuromuscular activation, and myokine signaling, which may augment lymphatic drainage and enhance vascular function. The strengthening of the pelvic floor, in conjunction with enhanced regional blood flow, fosters neurovascular adaptations that can lead to improved sexual function.

These interrelated mechanisms underscore the comprehensive nature of rehabilitation, highlighting the interdependence of musculoskeletal, lymphatic, and sexual health in individuals who have survived gynecologic cancer. Clinically, the empirical findings endorse the execution of personalized, multimodal rehabilitation programs as a standard component of care, meticulously tailored to the specific cancer type, treatment phase, and functional impairments of the patient. Such programs have the potential to optimize muscular strength, prevent or alleviate lymphedema, and enhance sexual health, consequently improving overall QoL, promoting independence, and fostering long-term survivorship.

Nevertheless, substantial deficiencies persist. The majority of studies are constrained by limited sample sizes, diverse protocols, and abbreviated follow-up durations. Future investigations should prioritize large-scale randomized controlled trials, mechanistic studies aimed at elucidating biological pathways, and the establishment of standardized outcome measures to facilitate inter-study comparisons. The incorporation of digital health monitoring, tele-rehabilitation, and patient-centered exercise prescriptions may further augment adherence and efficacy. Addressing these deficiencies will facilitate the formulation of evidence-based guidelines for comprehensive, multimodal rehabilitation in women afflicted with gynecologic cancers, ultimately advancing the domain of survivorship care.

Beyond its well-recognized therapeutic effectiveness, radiotherapy, especially HDR vaginal brachytherapy, remains a cornerstone in the treatment of gynecologic cancers in both curative and adjuvant contexts. Nevertheless, growing evidence indicates that these interventions are linked to clinically meaningful late adverse effects that extend beyond disease control and significantly influence long-term quality of life. Among the most frequent and consequential complications are vaginal toxicity and sexual dysfunction. Long-term follow-up studies have shown that HDR brachytherapy can result in various vaginal toxicities, including fibrosis, stenosis, mucosal thinning, and diminished tissue elasticity, with some effects persisting or progressing over time. Delishaj et al. (69) highlighted that vaginal toxicity continues to represent a notable late complication even years after treatment, underscoring the importance of ongoing monitoring and supportive interventions. These anatomical changes are often associated with functional consequences such as dyspareunia and decreased sexual activity, which can substantially impair overall well-being.

More recent clinical research further emphasizes the broader implications of brachytherapy on patient-reported outcomes. Facondo et al. (70) found that even short-course adjuvant HDR vaginal brachytherapy may adversely affect several quality-of-life domains, particularly those related to sexual health. Patients commonly describe diminished sexual desire, pain during intercourse, and emotional distress stemming from treatment-related changes. Such findings highlight the necessity of evaluating survivorship concerns alongside oncologic effectiveness when determining therapeutic approaches. Earlier investigations, including the study by Quick et al. (71), similarly demonstrated that intracavitary vaginal brachytherapy can lead to observable reductions in sexual function, even among women with early-stage disease. These outcomes are multifactorial, arising from both direct radiation-induced tissue injury and secondary psychosocial influences, reflecting the complex nature of treatment-related sexual dysfunction. Collectively, the available evidence underscores the need to systematically address brachytherapy-associated toxicities, particularly those affecting sexual health, in both clinical care and research settings. Enhancing awareness of these complications and proactively incorporating preventive and rehabilitative measures, such as vaginal dilator use, pelvic floor therapy, and structured sexual counseling, may help reduce their long-term burden. Ultimately, integrating functional and quality-of-life outcomes into treatment planning and follow-up strategies is essential for delivering comprehensive, patient-centered care in gynecologic oncology.
